# Assessment of drug resistance associated genetic diversity in Mauritanian isolates of *Plasmodium vivax* reveals limited polymorphism

**DOI:** 10.1186/s12936-018-2548-2

**Published:** 2018-11-08

**Authors:** Jemila Mint Deida, Yacoub Ould Khalef, Emal Mint Semane, Mohamed Salem Ould Ahmedou Salem, Hervé Bogreau, Leonardo Basco, Ali Ould Mohamed Salem Boukhary, Rachida Tahar

**Affiliations:** 1 Unité de Recherche Génome et Milieux (JEAI), Faculté des Sciences et Techniques, Université de Nouakchott Al-Aasriya, Nouveau campus universitaire, BP 5026 Nouakchott, Mauritania; 2 Unité Mixte de Recherche 216 Mère et enfant face aux infections tropicales, Institut de Recherche pour le Développement (IRD), Faculté de Pharmacie, Université Paris Descartes, 4 avenue de l’Observatoire, 75270 Paris Cedex 06, France; 3 Service de Pédiatrie, Centre Hospitalier Mère et Enfant (CHME), Nouakchott, Mauritania; 4 Centre de santé de Teyarett, Nouakchott, Mauritania; 5 Unité de Parasitologie et d’Entomologie, Institut de Recherche Biomédicale des Armées, IHU-Méditerranée Infection, Marseille, France; 6 Aix Marseille Univ, IRD, AP-HM, SSA, VITROME, IHU-Méditerranée Infection, Marseille, France; 7 Centre National de Référence du Paludisme, Institut Hospitalo-Universitaire (IHU) Méditerranée Infection, Marseille, France

**Keywords:** Malaria, Sahara, Drug resistance, Chloroquine, Sulfadoxine–pyrimethamine, Artemisinin

## Abstract

**Background:**

*Plasmodium vivax* is the predominant malaria species in northern Mauritania. Molecular data on *P. vivax* isolates circulating in West Africa are scarce. The present study analysed molecular markers associated with resistance to antifolates (*Pvdhfr* and *Pvdhps*), chloroquine (*Pvmdr1*), and artemisinin (*Pvk12*) in *P. vivax* isolates collected in two cities located in the Saharan zone of Mauritania.

**Methods:**

Blood samples were obtained from *P. vivax*-infected patients recruited for chloroquine therapeutic efficacy study in 2013 and febrile patients spontaneously consulting health facilities in Nouakchott and Atar in 2015–2016. Fragments of *Pvdhfr* (codons 13, 33, 57, 58, 61, 117, and 174), *Pvdhps* (codons 382, 383, 512, 553, and 585), *Pvmdr1* (codons 976 and 1076) and *Pvk12* (codon 552) genes were amplified by PCR and sequenced.

**Results:**

Most of the isolates in Nouakchott (126/154, 81.8%) and Atar (44/45, 97.8%) carried the wild-type *Pvdhfr* allelic variant (IPFSTSI). In Nouakchott, all mutants (28/154; 18.2%) had double *Pvdhfr* mutations in positions 58 and 61 (allelic variant IPF**RM**SI), whereas in Atar only 1 isolate was mutant (S117N, allelic variant IPFST**N**I). The wild-type *Pvdhps* allelic variant (SAKAV) was found in all tested isolates (Nouakchott, n = 93; Atar, n = 37). Few isolates in Nouakchott (5/115, 4.3%) and Atar (3/79, 3.8%) had the mutant *Pvmdr1* allele 976F or 1076L, but not both, including in pre-treatment isolates obtained from patients treated successfully with chloroquine. All isolates (59 in Nouakchott and 48 in Atar) carried the wild-type V552 allele in *Pvk12*.

**Conclusions:**

Polymorphisms in *Pvdhfr*, *Pvdhps*, *Pvmdr1*, and *Pvk12* were limited in *P. vivax* isolates collected recently in Nouakchott and Atar. Compared to the isolates collected in Nouakchott in 2007–2009, there was no evidence for selection of mutants. The presence of one, but not both, of the two potential markers of chloroquine resistance in *Pvmdr1* in pre-treatment isolates did not influence the clinical outcome, putting into question the role of *Pvmdr1* mutant alleles 976F and 1076L in treatment failure. Molecular surveillance is an important component of *P. vivax* malaria control programme in the Saharan zone of Mauritania to predict possible emergence of drug-resistant parasites.

## Background

Both *Plasmodium falciparum* and *Plasmodium vivax* are endemic in Mauritania with approximately 300,000 malaria cases reported in 2017 [1]. In the northern Saharan zone of Mauritania, particularly in Nouakchott, the capital city of the country, and Atar, the provincial capital of Adrar region, malaria transmission is seasonal, and *P. vivax* has been shown to be the main causative species of malaria parasite [2–4]. Infections caused by *P. vivax* have been considered for a long time as benign. Recently, several prospective studies recognized that *P. vivax* may be responsible for significant morbidity and even severe disease leading to mortality in endemic areas [5]. In the absence of an effective malaria vaccine, the use of insecticide-impregnated bed nets and antimalarial drug administration remain the only ways to prevent or treat the disease and reduce the probability of transmitting the parasite to *Anopheles* mosquitoes [6]. However, in many malaria-endemic countries, including Africa, the control strategy based on anti-malarial drugs has been facing the problem of parasite strains resistant to chloroquine, amodiaquine, antifolates, and mefloquine. In some countries, artemisinin resistance, including resistance to artemisinin-based combination therapy (ACT) dihydroartemisinin–piperaquine, has also emerged and spread [7–10]. To prevent, delay, and overcome the emergence of multidrug-resistant parasites, ACT has been recommended since 2001 for the treatment of uncomplicated malaria in endemic countries [11].

The mechanisms of resistance to antimalarial drugs, particularly in *P. falciparum*, have been studied for decades, but for some drugs, the mechanisms have not been fully elucidated. At least seven *P. falciparum* genes have been associated with drug resistance, namely *P. falciparum* chloroquine resistance transporter (*Pfcrt*) [12, 13], *P. falciparum* multi-drug resistance 1 (*Pfmdr1*) [14–16], *P. falciparum* dihydrofolate reductase (*Pfdhfr*) [17–19], *P. falciparum* dihydropteroate synthase (*Pfdhps*) [19–22], *P. falciparum* cytochrome *b* (*Pfcytb*) [23, 24] and *P. falciparum* sodium/hydrogen exchanger 1 (*Pfnhe*-1) [25, 26]. More recently, several mutations in the Kelch propeller domain of PF3D7_1343700 (*Pfk*13) were proposed to be directly responsible for artemisinin resistance in Cambodian isolates [27]. In addition to single nucleotide polymorphisms (SNPs), gene copy number may also be associated with resistance to certain drugs [14, 16].

In *P. vivax*, orthologous genes have been identified. Mutations in *dhfr* and *dhps* confer resistance to pyrimethamine and sulfadoxine, respectively, in both *P. falciparum* and *P. vivax* [22, 28–30]. Mutations in *Pfcrt* gene are associated with chloroquine resistance in *P. falciparum* whereas in *P. vivax* the role of *Pvcrt-o* (PfCRT-like protein, *P. vivax* orthologue also called *pvcg10*) mutations, insertion, gene copy number, or expression levels in conferring chloroquine resistance is not yet clear [31–36]. In *P. vivax*, mutations in *Pvmdr1* gene may be associated with resistance to chloroquine and amodiaquine [33, 37, 38].

The first molecular surveillance of *P. vivax* in Mauritania was conducted with isolates collected in 2007–2009 in Nouakchott, the capital city [3]. The present study is a follow-up molecular surveillance of *P. vivax* resistance in Nouakchott and Atar, both of which are situated in the Saharan zone of Mauritania, using *P. vivax* isolates collected between 2013 and 2016. As in the earlier study [3], polymorphisms in *Pvdhfr*, *Pvdhps*, and *Pvmdr1* were analysed in the present study. In addition, *P. vivax* kelch propeller domain located on chromosome 12 (*Pvk12*), which was shown to be homologous to *Pfk13* in 2015, was analysed in the present study [39]. *Pvcrt-o* gene was not sequenced in this study because there is no association between *Pvcrt-o* mutations and chloroquine resistance [31, 32].

## Methods

### Patients and blood sample collection

**Fig. 1:**
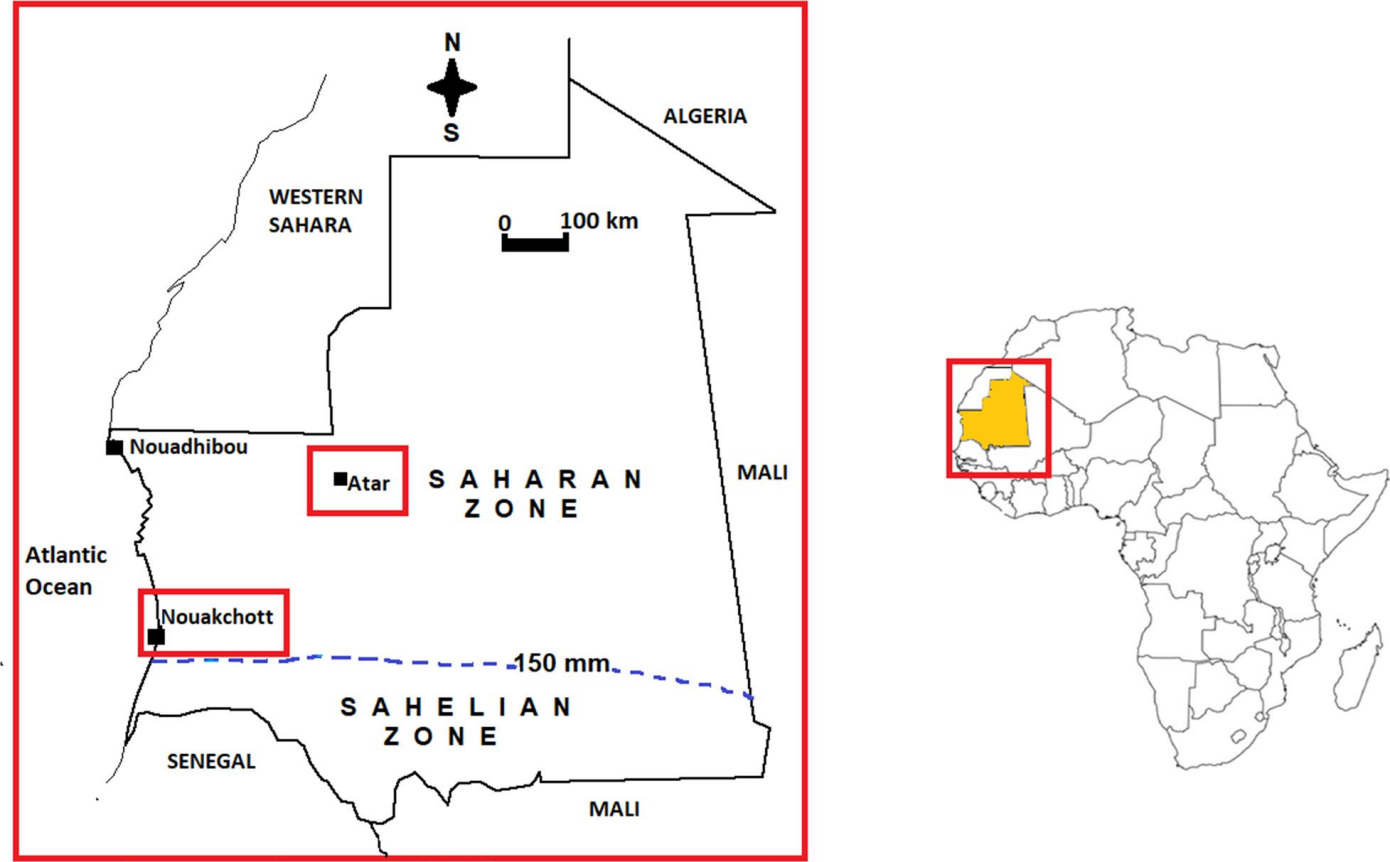
Map of Mauritania showing the study sites. The dotted line denotes 150 mm/year isohyet which indicates the southern limit of the Saharan zone

Blood samples used in the present study were collected from *P. vivax*-infected patients recruited for chloroquine therapeutic efficacy study conducted in Nouakchott and Atar in 2013 [40] and febrile patients spontaneously consulting health centres and hospitals and screened for malaria using rapid diagnostic test for malaria (SD Bioline *P. falciparum* histidine-rich protein 2 and *P. vivax* plasmodial lactate dehydrogenase antigen rapid diagnostic test; Standard Diagnostics, Inc., Yongin, Republic of Korea) in Nouakchott and Atar in 2015–2016 (Fig. 1). Two or three drops of fingerprick capillary blood (approximately 150–200 µL) were spotted directly on Whatman 3MM blotting paper (GE Healthcare Life Sciences, Bucks, UK), dried, and stored in individual sealed plastic bag at − 20 °C until use.

### PCR and sequencing of drug resistance genes

Parasite DNA was extracted from Whatman^®^ 3MM papers by Chelex^®^-saponin method [41]. *Plasmodium* species was identified by nested PCR targeting cytochrome *b* gene [42]. The presence of *Pvk12* mutation (V552I) observed in Cambodian *P. vivax* isolates was analysed using PCR protocol described by Popovici et al. [39].

Mutations associated with drug resistance in *Pvdhfr*, *Pvdhps*, and *Pvmdr1* genes were investigated using slightly modified nested PCR protocols described earlier [3]. The sequences of specific primers are presented in Table 1. The reaction mixture for the primary PCR amplification consisted of 10 µL of DNA template, 0.2 mM dNTP, 1.5 mM MgCl_2_, 0.25 μM of primer pairs, 1× reaction buffer and 2.5 U/μL *Taq* DNA polymerase (Bioline, France, Meridian Bioscience Europe, Paris, France) in a final volume of 50 μL.

**Table 1:** Primers used to amplify *Pvdhfr*, *Pvdhps*, *Pvmdr1*, and *Pvk12*

Gene	Primer sequences	Amplicon size (bp)^a^
*Pvdhfr*		
1st PCR	Forward: 5′-ACCCTTCCATAGGGAGTCCACTT-3′	961
	Reverse: 5′-CGCATTGCAGTTCTCCGAA-3′	
2nd PCR	Forward: 5′-CCCCACCACATAACGAAGTAG-3′	632
	Reverse: 5′-GCCGTTGATCCTCGTGAAG-3′	
*Pvdhps*		
1st PCR	Forward: 5′-GGAAGCCATTCGCTCAACTTATAA-3′	970
	Reverse: 5′-CGTCAGTTTACCCTCCCCGTT-3′	
2nd PCR	Forward: 5′-GATGGCGGTTTATTTGTCGAT-3′	767
	Reverse: 5′-GCCTCCCCGCTCATCAGTCT-3′	
*Pvmdr1*		
1st PCR	Forward: 5′-GCGAACTCGAATAAGTACTCCCTCTA-3′	762
	Reverse: 5′-GGCGTAGCTTCCCGTAAATAAA-3′	
2nd PCR	Forward: 5′-GGATTGCTGTCAGCACATATTAACA-3′	547
	Reverse: 5′AGAGGGATTTCATAAAGTCATCCACT-3′	
*Pvk12*		
1st PCR	Forward: 5′-ATCCAACAGCATTTCCAACT-3′	2108
	Reverse: 5′-CAATTAAAACGGAATGTCCA-3′	
2nd PCR	Forward: 5′-ACCACGTGACGAGGGATAAG-3′	1015
	Reverse: 5′-AAAACGGAATGTCCAAATCG-3′	

^a^Primer sequences and amplicon sizes were from Mint Lekweiry et al. [3] for *Pvdhfr*, *Pvdhps* and *Pvmdr1* and Popovici et al. [39] for *Pvk12*

Nested PCR amplifications were performed using a thermal cycler (Eppendorf 950000015 Mastercycler Gradient Thermal Cycler; Hinz GmbH, Hamburg, Germany). The following program was used to amplify *Pvdhfr*, *Pvdhps*, and *Pvmdr1* genes: initial denaturation at 95 °C for 5 min followed by 45 cycles of denaturation at 95 °C for 1 min, annealing at 53 °C for 1 min and elongation at 72 °C for 1 min, and the final elongation step of 72 °C for 10 min. For the secondary amplification, 2 μL of the primary PCR product was used as DNA template. The thermal cycling program and PCR mixture were identical to those of the primary PCR, except for the annealing temperature which was set at 65 °C and the volume of DNA template.

The products of the secondary PCR were visualized by electrophoresis on a 1% agarose gel. The PCR products were purified using Wizard^®^ SV gel and PCR clean-up system (Promega Corp, Fitchburg, WI) according to the manufacturer’s instructions. DNA sequencing of the PCR products was performed by Eurofins Genomics (Les Ulis, France) using BigDye Terminator v3.1 cycle sequencing kit (Applied Biosystems Courtaboeuf, France) and ABI 3730XL sequencer. Sequences were read using Chromas DNA sequencing software (Technelysium Pty Ltd., South Brisbane, Australia) and aligned using Clustal X2 software (Conway Institute, University College Dublin, Dublin, Ireland) to search for SNPs. Polymorphisms were identified in both forward and reverse strands by comparing the sequences to the reference sequences of *Pvdhfr* (GenBank accession no X98123), *Pvdhps* (GenBank accession no AY186730), *Pvmdr1* (Genbank accession no AY618622), and *Pvk12* (PVX_083080).

## Results

Among blood samples collected in Nouakchott and confirmed positive by rapid diagnostic test, the molecular markers of drug resistance were analysed for the following numbers of isolates: 154 for *Pvdhfr*, 93 for *Pvdhps*, 115 for *Pvmdr1*, and 59 for *Pvk12* (Table 2). For *Pvdhfr*, a large majority of isolates (126/154, 81.8%) had wild-type alleles at positions 13, 33, 57, 58, 61, 117, and 173 (wild-type allelic variant IPFSTSI). Twenty-eight isolates (18.2%) were double mutants with 58R and 61M (mutant allelic variant IPF**RM**SI). All isolates (93/93, 100%) had the wild-type *Pvdhps* allelic variant (SAKAV). Sequencing of the *Pvmdr1* gene revealed that a large majority of isolates (110/115, 95.7%) were of wild-type at positions 976 and 1076, while only 5 isolates (4.3%) had the mutant allele 976F. None of the isolates carried the mutant 1076L allele. All isolates (59/59, 100%) collected in Nouakchott had the wild-type allele V552 in *Pvk12* (Table 2).

**Table 2:** Prevalence of *Pvdhfr*, *Pvdhps*, *Pvmdr1*, and *Pvk12* allelic variants in Nouakchott and Atar, Mauritania

Gene	Allelic variant^a^	Nouakchott n/N (%)	Atar n/N (%)
*Pvdhfr*	IPFSTSI (wild-type)	126/154 (81.8)	44/45 (97.8)
	IPF**RM**SI	28/154 (18.2)	0
	IPFST**N**I	0	1/45 (2.2)
*Pvdhps*	SAKAV (wild-type)	93/93 (100)	37/37 (100)
*Pvmdr1*	YF (wild-type)	110/115 (95.7)	76/79 (96.2)
	**F**F	5/115 (4.3)	1/79 (1.3)
	Y**L**	0	2/79 (2.5)
*Pvk12*	V552 (wild-type allele)	59/59 (100)	48/48 (100)
	552I	0	0

*n* number of isolates with the given allelic variant *N* total number of isolates analysed

^a^Allelic variants are based on the following codons: 13, 33, 57, 58, 61, 117, and 174 for *Pvdhfr*; 382, 383, 512, 553, and 585 for *Pvdhps*; and 976 and 1076 for *Pvmdr1*. The amino acid substitution V552I in *Pvk12* was suggested to be a potential marker for artemisinin-resistant *P. vivax* in Cambodia [39]

With respect to *P. vivax*-infected blood samples collected in Atar and confirmed to be positive by rapid diagnostic test for malaria, 45, 37, 79, and 48 amplification products were analysed for *Pvdhfr*, *Pvdhps*, *Pvmdr1* and *Pvk12*, respectively (Table 2). For *Pvdhfr*, most isolates (44/45, 97.8%) exhibited wild-type alleles in codons 13, 33, 57, 58, 61, 117, and 173, giving the allelic variant IPFSTSI. Only one isolate (2.2%) carried a single S117N mutation (mutant allelic variant IPFST**N**I). For *Pvdhps*, all isolates (37/37, 100%) were of wild-type allelic variant (SAKAV) at positions 382, 383, 512, 553 and 585. Most isolates (76/79, 96.2%) carried wild-type Y976 and F1076 codons in the *Pvmdr1* gene. Only three isolates (3/79, 3.8%) had 976F (n = 1 isolate) or 1076L (n = 2) mutant alleles. As in Nouakchott, all isolates (48/48, 100%) were characterized to carry the wild-type V552 allele in *Pvk12* (Table 2).

## Discussion

In Mauritania, the treatment of uncomplicated malaria had been based on chloroquine and sulfadoxine–pyrimethamine (SP) as the first- and second-line drugs until 2006, respectively. Following the emergence and spread of chloroquine-resistant *P. falciparum* in West Africa [43], the Mauritanian health authorities adopted, in 2006, a new therapeutic strategy for the management of malaria cases based on ACT (using either artesunate–amodiaquine or artemether–lumefantrine) as the first-line treatment for all malaria cases without distinction of *Plasmodium* species. This national drug policy was adopted to conform to the WHO guidelines for African countries and was not a decision based on previous clinical studies demonstrating chloroquine-resistant *P. falciparum* in Mauritania.

Unlike *P. falciparum*, which is the predominant *Plasmodium* species in Africa, molecular data on *P. vivax* are scarce to non-existent in most West African countries. The Saharan zone of Mauritania is an exception [44, 45]. The results of the present study showed that mutations in *Pvdhfr*, *Pvdhps*, *Pvmdr1* and *Pvk12* occur in some *P. vivax* isolates collected recently in the Saharan zone of Mauritania. It was also observed that in *Pvdhfr*, a marker known to accumulate mutations at specific codons and increase the level of resistance to pyrimethamine, the number of mutations in a mutant isolate was limited to two, and the key *Pvdhfr* S117N substitution was observed in a single isolate from Atar. These results are in general agreement with the previous study conducted in isolates collected in 2007–2009 in Nouakchott [3]. In that study, only 12% (10 of 86) of isolates were mutants carrying double mutations S58R and S117N, and, as in the present study, none of the isolates analysed earlier (n = 94) had mutations in *Pvdhps*. For more than a decade, the use of SP has been restricted to pregnant women for intermittent preventive treatment (IPTp) in Mauritania, as well as in children for seasonal malaria chemoprevention (amodiaquine + SP) in some African countries in the Sahelian zone. Both of these preventive strategies are directed against *P. falciparum*. The use of SP to treat *P. vivax* malaria is not recommended due to its inherent resistance or lower susceptibility to antifolates [46]. In practice, some pregnant Mauritanian women under IPTp in the Saharan zone are exposed to the risk of *P. vivax* infection. Although prospective clinical studies would be required to confirm the benefit of IPTp even against *P. vivax*, molecular data tend to support SP efficacy against *P. vivax*.

The earlier molecular study in Nouakchott showed that most isolates (75/103, 73%) had Y976 wild-type *Pvmdr1* allele, as in the present study (110/115, 95.7%) [3]. However, that study showed a high proportion (98%) of mutant F1076L allele in *Pvmdr1*, whereas in the present study, mutation was absent in codon 1076. The origin of this discrepancy is unknown. This observation calls for further molecular monitoring in Nouakchott. *Pvmdr1* Y976F and F1076L mutations were suggested to be associated with chloroquine and amodiaquine resistance [33, 37, 38]. However, several in vitro and clinical studies have failed to confirm the association between *Pvmdr1* mutations and drug resistance [47–53]. In the present study, among 115 and 79 isolates with *Pvmdr1* sequences in Nouakchott and Atar, 51 and 48 were pre-treatment blood samples from patients treated with chloroquine in 2013, respectively. In many patients enrolled in chloroquine therapeutic efficacy studies, parasitaemia was cleared on or before day 3, and none of the patients had parasitaemia on day 7 [40]. The outcome was adequate clinical and parasitological response in all patients on day 28. These results suggest that the presence of one of the two mutant *Pvmdr1* alleles (i.e., 976F or 1076L, but not both) is not associated with chloroquine treatment failure in Nouakchott and Atar. It remains unclear whether both 976F and 1076L are required for chloroquine treatment failure since none of the isolates analysed in the present study carried the double mutations in *Pvmdr1*. Further clinical and molecular studies are required to assess the relevance of *Pvmdr1* mutations in chloroquine resistance, in particular in African countries, such as Ethiopia, where chloroquine-resistant *P. vivax* occurs [54–57].

Artemisinin-resistant *P. falciparum* occurs in Southeast Asia, and resistance seems to be conferred by specific mutations in the propeller domain of Kelch 13 (K13; PF3D7_1343700) [27]. In *P. vivax*, *Pvk12* was identified as the homologous gene of *Pfk13* [39]. At present, there is no molecular indication that *Pvk12* undergoes a high degree of mutations as its *P. falciparum* homolog, even in Southeast Asia [39, 58–60]. The sequence data of Mauritanian *P. vivax* isolates also indicate the absence of polymorphism in the *Pvk12* gene fragment that was amplified and analysed. Moreover, clinical and in vitro studies carried out so far do not suggest artemisinin resistance in *P. vivax*. Further molecular surveillance is warranted to anticipate the possible emergence of artemisinin-resistant *P. vivax* around the world.

## Conclusions

Few Mauritanian *P. vivax* isolates were characterized to carry mutations in *Pvdhfr*, *Pvdhps*, *Pvmdr1*, and *Pvk12* markers. The molecular data are in general agreement with the high clinical efficacy of chloroquine previously demonstrated in Nouakchott and Atar and with an earlier study on isolates collected in 2007–2009 in Nouakchott, suggesting that mutations in these molecular markers of drug resistance are not being selected. Due to the unavailability of chloroquine in the official drug outlets of the country, *P. vivax* infections have been treated with ACT over more than a decade. This may explain, at least in part, the absence of selection of mutant *P. vivax* parasites. Molecular surveillance of *P. vivax* is an important component of malaria control in northern Mauritania where this parasite species is predominant.

## Abbreviations

ACT: arteminisin-based combination therapy
IPTp: intermittent preventive treatment in pregnancy
*Pfcrt*: *P. falciparum* chloroquine resistance transporter
*Pfcytb*: *P. falciparum* cytochrome *b*
*Pfdhfr*: *P. falciparum* dihydrofolate reductase
*Pfdhps*: *P. falciparum* dihydropteroate synthase
*Pfk13*: *P. falciparum* Kelch 13
*Pfmdr1*: *P. falciparum* multi-drug resistance 1
*Pfnhe*-*1*: *P. falciparum* sodium/hydrogen exchanger 1
*Pvcrt*-*o*: *Plasmodium vivax* chloroquine resistance transporter orthologue
*Pvdhfr*: *Plasmodium vivax* dihydrofolate reductase
*Pvdhps*: *Plasmodium vivax* dihydropteroate synthase
*Pvk12*: *Plasmodium vivax* Kelch 12
*Pvmdr1*: *Plasmodium vivax* multidrug resistance 1
SNP: single nucleotide polymorphism
SP: sulfadoxine–pyrimethamine
WHO: World Health Organization
